# Safety and Immunogenicity of Anti-SARS-CoV-2 Booster Dose in Patients with Chronic Liver Disease

**DOI:** 10.3390/jcm12062281

**Published:** 2023-03-15

**Authors:** Valentina Cossiga, Mario Capasso, Maria Guarino, Ilaria Loperto, Stefano Brusa, Francesco Maria Cutolo, Maria Rosaria Attanasio, Raffaele Lieto, Giuseppe Portella, Filomena Morisco

**Affiliations:** 1 Diseases of the Liver and Biliary System Unit, Department of Clinical Medicine and Surgery, University of Naples “Federico II”, 80131 Naples, Italy; 2 UOC Epidemiologia e Prevenzione e Registro Tumori, ASL Napoli 1 Centro, 80148 Naples, Italy; 3 Department of Translational Medical Science, University of Naples “Federico II”, 80131 Naples, Italy

**Keywords:** COVID-19, SARS-CoV-2, chronic liver disease, vaccine, cirrhosis

## Abstract

The low response to vaccines is a well-known problem in cirrhosis. We evaluated the safety and immunogenicity of booster doses in patients with chronic liver disease (CLD), comparing the humoral response in cirrhotic vs. non-cirrhotic patients, and the impact of different factors on immune response. From September 2021 to April 2022, outpatients with CLD who completed the primary vaccination course and the booster dose against SARS-CoV-2 were enrolled. Blood samples were collected after second and third doses for detecting anti-spike protein IgG. We enrolled 340 patients; among them, 91 subjects were cirrhotic. After primary vaccination course, 60 (17.6%) patients did not develop a positive antibody titer, without significant differences between cirrhotic and non-cirrhotic patients (*p* = 0.076); most of them (88.3%) developed it after booster dose. At multivariable analysis, factors associated with higher humoral response after booster dose were only porto-sinusoidal vascular disorder (*p* = 0.007) as an etiology of CLD and the use of the mRNA-1273 vaccine (*p* = 0.001). In conclusion, in patients with CLD, a booster dose against SARS-CoV-2 induces an excellent immunogenicity and leads to an adequate antibody response. Cirrhosis is not associated with a worse humoral response, compared to patients with non-cirrhotic CLD.

## 1. Introduction

Since December 2019, SARS-CoV-2 (severe acute respiratory syndrome-related coronaVirus) infection [1] rapidly spread worldwide, with about 651,000,000 people infected and 6,600,000 dead [2].

In Europe, Italy was one of the most affected countries [3]. For this reason, on 27 December 2020, after EMA (European Medicines Agency) and AIFA (Agenzia Italiana del Farmaco) vaccine approvals [4], vaccination against SARS-CoV-2 began in Italy with the administration of two doses (primary course) [4,5]. Subsequently, a third dose, as a booster dose, has been recommended [6].

According to specific criteria (elder people, patients with immunodeficiency, neoplasia, cardiovascular, or pulmonary or chronic kidney or liver disease, or patients with multiple comorbidities) associated with a higher risk of severe illness or hospitalization due to Coronavirus disease 2019 (COVID-19), people were classified in at-risk categories [7,8,9] and were prioritized for vaccine administration.

It is well-known that patients with chronic liver disease (CLD) have an immune-dysregulation associated with several abnormalities in innate and adaptive components of immune system, leading to poor immunological response to infection and vaccines [10,11].

Patients with CLD have high overall mortality for COVID-19 (32%), increasing with Child–Pugh class (patients with CLD without cirrhosis, as well as general population: 8%, Child–Pugh A 19%, Child–Pugh B 35%, and Child–Pugh C 51%) [12]. Additionally, our previous study [13] demonstrated that liver transplant recipients with COVID-19 were significantly more frequently symptomatic, with a higher rate of hospitalization and mortality. For these reasons, patients with CLD were considered “frail patients” and have been prioritized in the timeline schedule of the National Vaccination Program. Nonetheless, very poor data are available regarding the safety and immunogenicity of SARS-CoV-2 vaccines in patients with CLD, and detailed information on the immune response after vaccination of cirrhotic patients against SARS-CoV-2 is needed.

In this scenario, the main aim of the present study is to evaluate the safety and immunogenicity of SARS-CoV-2 vaccines in patients with CLD. The secondary endpoint is the comparison of humoral response between cirrhotic and non-cirrhotic patients and the impact of different factors on the immune response.

## 2. Materials and Methods

### 2.1. Study Design and Target Population

This single-center prospective study was conducted on patients with CLD, enrolled during their outpatient follow-up at the “Diseases of the Liver and Biliary System” Unit (University of Naples, “Federico II”).

From September 2021 to April 2022, all consecutive patients with CLD referred to our unit who completed primary vaccination course against SARS-CoV-2 and who received booster dose were enrolled.

Exclusion criteria were patients who refuse consent, patients <18 years old, liver transplanted (LT) subjects, patients who refuse vaccination, incomplete primary vaccination course, and patients with previous and subsequent SARS-CoV-2 infection between primary vaccination course and booster dose.

### 2.2. Data Collection

We collected clinical and biochemical data from medical records up to three months before enrollment: age, gender, body mass index (BMI), etiology of chronic liver disease, comorbidities, drug therapies (in particular immunosuppressive treatment), portal hypertension status, and presence of hepatocellular carcinoma (HCC).

A questionnaire was submitted to all the enrolled patients to investigate the status of primary vaccination course and booster dose, the type of vaccine and side effects (local symptoms such as redness, swelling, or pain at the injection site, regional lymphadenopathy, or systemic symptoms such as fever, chills, headache, arthralgia, myalgia, asthenia, nausea, vomiting, diarrhea), and other uncommon adverse reaction, or anaphylaxis.

### 2.3. Blood Sampling and Imaging Acquisition

Blood samples were collected at 2 time-points: 12–16 weeks after primary vaccination course and 12–16 weeks after booster dose.

Cirrhosis was evaluated performing transient liver elastometry using Fibroscan^®^ (Echosens, Paris, France), a non-invasive technique with high sensitivity and specificity for the diagnosis of liver fibrosis [14,15]. Liver stiffness value considered for diagnosis of cirrhosis was ≥12.5 kPa [16,17,18]. Fibroscan^®^ was performed at enrollment in fasting patients for at least five hours; the exam was considered valid if at least 10 measurements were made with the same probe and if InterQuartile range/median ratio of liver stiffness was <30% [19].

Portal hypertension was assessed considering the direct presence of gastroesophageal varices, portal hypertensive gastropathy, and/or indirect signs of clinically significant portal hypertension (CSPH) (liver stiffness > 25 kPa, splenomegaly, and thrombocytopenia).

### 2.4. Quantification of Humoral Response

Freshly collected blood in clot activator and gel tube was centrifuged at 4500 rpm for 10 min. The sera were separated and stored at −20 °C for the subsequent analysis. Collected samples were analyzed for quantitative determination of anti-trimeric spike protein-specific IgG antibodies to SARS-CoV-2 with indirect chemiluminescence immunoassay “LIAISON SARS-CoV-2 TrimericS IgG assay (DiaSorin S.p.A. Saluggia, Italy) [20], and antibody titer was expressed in binding antibody unit (BAU)/mL. Results were considered positive if patients had antibody titer ≥33.8 BAU/mL.

### 2.5. Statistical Analysis

The Shapiro–Wilks test was used to assess the conformity of the parameters to the normal distribution, while the continuous variables were expressed as median and inter-quartile range (IQR), and the categorical variables were described as absolute frequency and percentage.

The descriptive statistics of the variables under study were performed using the Kruskal–Wallis test, the chi-square test, and Fisher’s exact test, as appropriate.

The correlation between continuous variables was investigated using Spearman’s rank correlation test.

Risk evaluation of non-response to vaccination was evaluated by coefficient and 95% confidence intervals (95% CI) by means of multivariate regression models, considering only those significant at the univariable analysis as independent variables. The continuous dependent variable considered was the antibody titer after the third dose. A *p*-value ≤ 0.05 was considered statistically significant. Stepwise forward regression method has been used to confirm the independent variables selected. Statistical analyses were performed using “STATA 15” statistical software (StataCorp, College Station, TX, USA).

## 3. Results

### 3.1. Characteristics of Population

A total of 340 outpatients with CLD who followed up at the “Diseases of the Liver and Biliary System” Unit (University of Naples, “Federico II”) were enrolled. A total of 187 (55%) subjects were male, and the median age at the time of enrollment was 64.32 ± 17.34 years. The general characteristics of the studied population are described in Table 1.

BNT162b2 was the anti-SARS-CoV-2 vaccine more frequently administered (67.06%), followed by mRNA-1273 (7.94%) and ChAdOx1-S (5.29%), while almost 20% of patients received heterologous vaccination.

A total of 91 (26.76%) patients were cirrhotic (Child–Pugh A 80.2%, B 17.5%, and C 2.2%), and 249 (73.23%) were non-cirrhotic. Cirrhotic patients were predominantly male (*p* = 0.02) and significantly younger than non-cirrhotic (*p* = 0.002). Clinically significant portal hypertension was present in 57 (62.2%) cirrhotic patients and in 5 (2%) non-cirrhotic patients (all of them with porto-sinusoidal vascular disease (PSVD)).

In cirrhotic patients, the more frequent etiologies were hepatitis C virus (HCV) infection, metabolic-associated fatty liver disease (MAFLD), and alcoholic liver disease (ALD), while in non-cirrhotic patients, hepatitis B virus (HBV) infection and autoimmune liver diseases were the main causes of CLD (*p* < 0.001).

Median liver stiffness in cirrhotic patients was higher than in non-cirrhotic: 19.25 ± 17.6 kPa vs. 5.8 ± 3.2 kPa (*p* < 0.001).

Cirrhotic patients had two or more comorbidities more often than non-cirrhotic ones (53.8% vs. 24.9%; *p* < 0.001); chronic obstructive pulmonary disease (COPD) (*p* = 0.001) and type II diabetes (*p* ≤ 0.001) were the most frequent.

Regarding vaccination, cirrhotic patients received more frequent BNT162b2 vaccines than non-cirrhotic patients (*p* = 0.049).

### 3.2. Humoral Response after Vaccination Course

**Figure 1:**
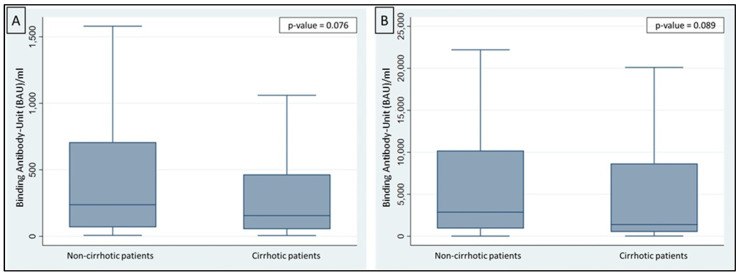
(**A**) Antibody titer in non-cirrhotic and in cirrhotic patients after primary vaccination course (*p*-value = 0.076). (**B**) Antibody titer in non-cirrhotic and in cirrhotic patients after booster dose (*p*-value = 0.089).

At the end of the primary vaccination course (2 doses), 60 patients (17.6%) did not develop a positive antibody titer, without differences between cirrhotic and non-cirrhotic groups (median antibody titer 157 ± 326 BAU/mL vs. 264 ± 592 BAU/mL; respectively 19.7% vs. 16.8%; *p* = 0.076) (Figure 1A). Most of them (53/60 patients; 88.3%) developed a positive serology titer after the booster dose, without differences between cirrhotic and non-cirrhotic (*p* = 0.089).

After the third dose, the median antibody titer was higher in both groups (1310 ± 6486 BAU/mL in cirrhotic vs. 2700 ± 9222 BAU/mL in non-cirrhotic patients, *p* = 0.089, Figure 1B). Only 7 (11.7%) patients showed a negative antibody titer (<33.8 BAU/mL), without statistically significant differences between cirrhotic and non-cirrhotic patients (2.7% vs. 2.5%). A total of three of the seven patients with negative humoral response after booster dose (42.8%) were taking immunosuppressive therapy at the time of vaccination.

Analyzing the antibody titer after the booster dose, we noticed that, although not statistically significant, the titer was two times higher in compensated vs. decompensated cirrhotic patients (2490 ± 9306 BAU/mL in Child A, 707.5 ± 10,510.7 BAU/mL in Child B and 530 ± 0 BAU/mL in Child C), as well as in patients without CSPH vs. those with CSPH (2619.5 ± 9280 BAU/mL vs. 1390 ± 6518 BAU/mL) and in patients without HCC vs. those with HCC (2619.5 ± 8936 BAU/mL vs. 1390 ± 10,910.5 BAU/mL).

### 3.3. Factors Associated with Positive Humoral Response

At univariable analysis, the factors associated with a positive humoral response after booster dose were younger age (*p* = 0.0098), PSVD as etiology (*p* = 0.005), none or a single comorbidity, rather than two or more comorbidities (*p* = 0.05), and the mRNA-1273 booster dose, compared with the BNT162b2 (*p* = 0.001).

At multivariable analysis, only the PSVD (*p* = 0.007) and mRNA-1273 (*p* = 0.001) vaccines were associated with a positive humoral response after booster dose (Table 2). The stepwise forward regression confirmed the results.

**Figure 2:**
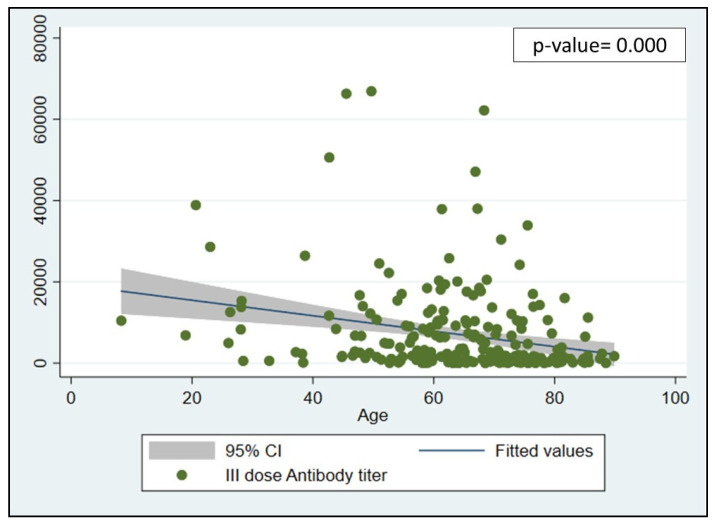
Scatterplot: correlation between age and antibody titer in patients with chronic liver disease. It shows an inverse relationship (Spearman rho = −0.28; *p* < 0.001). Abbreviation: CI (Confidence Interval).

Moreover, in the entire population, both in cirrhotic and non-cirrhotic patients, we found a trend of inverse correlation between age and antibody titers (Spearman rho = −0.28; *p* < 0.001), as shown in Figure 2.

### 3.4. Tolerability and Safety of COVID-19 Vaccines

The vaccination was well-tolerated. No major adverse events occurred in all enrolled patients. There were no significant differences between the primary course and the booster dose for all the reported side effects. Overall, mild local and systemic symptoms were registered in 11 (3.24%) and 36 (10.59%) patients, respectively. The most commonly reported side effects were local pain at injection site (36.1%), fatigue (12%), and low-graded fever (10%). These mild adverse events were higher in non-cirrhotic, compared to cirrhotic, patients (*p* = 0.007). None of the cirrhotic patients developed liver-related adverse event, such as acute on chronic liver failure or worsening of liver function.

## 4. Discussion

In this prospective study, we evaluated the safety and the immunogenicity of booster doses of anti-SARS-CoV-2 vaccines in a large cohort of patients with CLD. Even if, in the literature, there are many studies describing the immunological response to the primary course of anti-SARS-CoV-2 vaccination (two doses) in CLD patients, very few data are available after the administration of booster dose. Moreover, most of published studies compared the immune response of cirrhotic patients with a healthy control, showing a lower immunological response to anti-SARS-CoV-2 vaccines in cirrhotics [21,22,23].

Differently, in our study, we enrolled a cohort of patients with different degrees of CLD, aiming to compare humoral responses between cirrhotic and non-cirrhotic. The main results were the absence of a significant difference in the humoral response between the two groups and the ability of the booster dose to also induce a positive antibody titer in cirrhotic patients without a positive response to the primary vaccination course. Similar results were also observed in a prospective multicentric study conducted in China by Jingwen Ai et al. [24], showing that immunogenicity is similar in patients with non-cirrhotic CLD, compensated cirrhosis, and decompensated cirrhosis and confirming that immunologic response to SARS-CoV-2 vaccines in all these groups is lower than healthy population.

In more detail, we demonstrated that the majority of patients (53/60 subjects; 88.3%) with a negative titer after a primary vaccination course developed a positive and humoral response after the booster dose, confirming the efficacy and the importance of booster dose administration in this category. Similar results were obtained, in a large cohort (13,041 cirrhotic patients who received the third dose matched to 13,041 controls who received two doses) by John BV et al. [25], showing that the booster dose was associated to a significant reduction in the occurrence of COVID-19 infection, as well as in the hospitalization and mortality rates. Particularly, none of cirrhotic patients who received a third dose died to COVID-19 infection. Additionally, in our study, we showed that the antibody titer increased after the booster dose, mainly in non-cirrhotic patients, but also in cirrhotic ones. So, it is important to evaluate the kinetics of the humoral response over time, as shown by Iavarone M. et al. [23], demonstrating that there was a significant increase in antibody titer between the first and the second dose in cirrhotic patients, as well as in healthy controls. Similar results were also observed in a previous study by our group [11] in a large cohort of liver transplanted (LT) patients showing that many LT patients with a negative titer after primary dose showed a positive serology after the second dose. 

It is also important to underline that our knowledge on the concept of reduced humoral response in cirrhotic patients is mainly based on anti-influenza vaccination studies [26]. Nonetheless, this type of vaccination is completely different from COVID-19 vaccination, and the previous studies analyzed only a single timepoint after the vaccine for influenza. Therefore, our findings support recommendations from liver societies on booster dose administration in patients with CLD.

In our cohort, factors associated with a positive humoral response were the PSVD and mRNA-1273 vaccines. PSVD is a vascular liver disorder that occurs in the absence or lower grade of hepatocellular fibrosis, compared to other CLDs. Even if with the limit of the small sample size (only 9 patients with PSVD), it should not be surprising that these patients show a better immunological response to vaccine than CLD or cirrhotic patients, in which the fibrotic damage of the liver probably induces a reduction in the protein synthesis involved in the immune response, with subsequential dysregulation and lower antibody production [27].

According to the better response to mRNA-1273 vaccine administration, our results are in line with those by Iavarone M. et al. [23], concluding that anti-spike levels were significantly higher in those who received mRNA-1273 vaccine, compared to patients who received BNT162b2.

Interestingly, we found a trend of inverse correlation between age and antibody titers, with higher antibody titers in the younger population, compared to the older population. The main reason for this phenomenon should be referred to the better immunological status in young people related to the well-known immune senescence (i.e., an age-related dysregulation and decline of the immune system) with a weak response to vaccinations in older subjects [28].

Despite the fact that the analysis of the humoral response in cirrhotic subgroups (compensated vs. decompensated cirrhotic patients, patients without CSPH vs. those with CSPH, and patients without HCC vs. those with HCC) showed no statistical significance, we noticed that our results lean toward the differences between these subgroups. The smallness of sample size was the main determining factor in the analysis, and a statistic significance should be obtained with a larger sample, as reported in a prospective multicenter study [29], in which Jitao W. et al. described the humoral hyporesponsiveness to COVID-19 vaccination in decompensated cirrhotic patients (Child–Pugh scores B and C) and in HCC patients [30].

Finally, we showed that the vaccination was well-tolerated and that the booster dose was also safe in cirrhotic patients, without difference in compensated and decompensated subjects. Indeed, in our cohort, no serious adverse event occurred, most of the side effects we registered were mild, and none of cirrhotic patients developed decompensation after the vaccination. Moreover, any adverse event reported did not compromise the subsequent vaccine administration; therefore, we did not observe any patient dropout in the study.

The main strength of this study includes the evaluation of humoral response after booster dose in a large cohort of patients with CLD comparing the cirrhotic and non-cirrhotic patients, differently from the majority of the published studies comparing cirrhotic patients with healthy subjects.

Nonetheless, the main limitation of this study is the lack of evaluation of the cell-mediated immune response to vaccination. Additionally, for the detection of antibody titers, we used a kit that is considered the gold standard for the evaluation of the anti-S antibodies, the DiaSorin LIAISON immunoassay (sensitivity, 99.4%; specificity, 99.8%) [31].

## 5. Conclusions

The booster dose is safe and efficient in patients with CLD. Cirrhosis is not associated with a worse humoral response compared with patients with non-cirrhotic CLD. Some subgroups of cirrhotic patients (the ones with decompensated cirrhosis, with clinically significant portal hypertension, or with HCC) may have a reduced humoral response, but more data and a larger sample size need to be evaluated.

## Figures and Tables

**Table 1:** General characteristics of population and comparison between cirrhotic and non-cirrhotic groups.

General Characteristics	Overall Population (*n* = 340)	Cirrhotic	Non-Cirrhotic (*n* = 249)	*p*-Value
		(*n* = 91)		
Age, years, (median ± IQR)	64.32 ± 17.34	62.6 ± 19.32	68.02 ± 13.42	0.0001 *
<40, *n* (%)	28 (8.24)	2 (2.2)	26 (10.44)	
40–65, *n* (%)	144 (42.35)	31 (34.07)	113 (45.38)	0.002 **
>65, *n* (%)	168 (49.41)	58 (63.74)	110 (44.18)	
Gender, male, *n* (%)	187 (55)	59 (64.84)	128 (51.41)	0.028 **
BMI, Kg/m^2^, (median ± IQR)	26.6 ± 5.3	26.6 ± 5.1	26.4 ± 5	0.73 *
Smoker, *n* (%)	24 (7.06)	14 (15.38)	10 (4.02)	<0.001 **
Etiology of liver disease, *n* (%)				
HBV	104 (30.59)	20 (21.98)	84 (33.73)	
HCV	116 (34.12)	36 (39.56)	80 (32.13)	
MAFLD	50 (14.71)	17 (18.68)	33 (13.25)	<0.001 **
ALD	12 (3.53)	8 (8.79)	4 (1.61)	
Autoimmune	44 (12.94)	4 (4.4)	40 (16.06)	
PSVD	9 (2.65)	3 (3.3)	6 (2.41)	
Genetic disease	5 (1.47)	3 (3.3)	2 (0.8)	
HCC, *n* (%)	31 (9.12)	31 (34.07)	0 (0)	<0.001 **
Child–Pugh, *n* (%)				
Class A	73 (21.4)	73 (80.22)	-	
Class B	16 (4.7)	16 (17.58)	-	-
Class C	2 (0.59)	2 (2.2)	-	
MELD, (median ± IQR)	9 ± 4	9 ± 4	-	-
Portal hypertension, *n* (%)	62 (18.24)	57 (62.24)	5 (2.01)	<0.001 **
Liver stiffness, kPa, (median ± IQR)	6.4 ± 6	19.25 ± 17.6	5.8 ± 3.2	0.0001 *
Immunosuppressive therapy, *n* (%)	24 (7)	5 (5.49)	19 (7.6)	0.623 **
Steroids, *n* (%)	7 (2.06)	0 (0)	7 (2.81)	0.106 **
Comorbidities, *n* (%)				
Cardiovascular disease	48 (14.2)	16 (17.58)	32 (12.85)	0.267 **
Arterial hypertension	172 (50.59)	52 (57.14)	120 (48.19)	0.144 **
Type II diabetes	64 (18.82)	30 (32.97)	34 (13.65)	<0.001 **
Concomitant neoplasia	44 (12.94)	32 (35.16)	12 (4.82)	<0.001 **
History of previous neoplasia	26 (7.65)	7 (7.69)	19 (7.63)	0.985 **
Chronic kidney disease	22 (6.47)	7 (7.69)	15 (6.02)	0.580 **
COPD	18 (5.29)	11 (12.09)	7 (2.81)	0.001 **
Two or more comorbidities, *n* (%)	111 (32.65)	49 (53.85)	62 (24.9)	<0.001 **
Type of vaccination, *n* (%)				
BNT162b2	228 (67.06)	71 (78.02)	157 (63.05)	
mRNA-1273	27 (7.94)	4 (4.4)	23 (9.24)	0.049 **
ChAdOx1-S	18 (5.29)	5 (5.49)	13 (5.22)	
Heterologous vaccination	67 (19.71)	11 (12.09)	56 (22.49)	
Adverse events, *n* (%)				
Local	11 (3.24)	2 (2.2)	9 (3.61)	0.007 **
Systemic	36 (10.59)	2 (2.2)	34 (13.65)	

* Kruskal–Wallis test; ** Chi-square test/Fisher’s exact test. Abbreviation: IQR (InterQuartile range), BMI (Body mass index), HBV (Hepatitis B Virus), HCV (Hepatitis C Virus), MAFLD (metabolic-associated liver disease), ALD (alcoholic liver disease), PSVD (porto-sinusoidal vascular disorder), HCC (Hepatocellular carcinoma), MELD (Model for End-stage Liver Disease), kPa (kilopascal), COPD (Chronic obstructive pulmonary disease).

**Table 2:** Univariable and multivariable analysis on factors associated with positive serology to booster dose (dependent variable: positive titer).

Characteristics of Population	Univariate Analysis (95%CI)		Multivariate Analysis (95%CI)	
	Antibody Titer (Median ± IQR)	*p*-Value	Antibody Titer (Median ± IQR)	*p*-Value
Age, years (Spearman rho)	−0.28	<0.001 #	−68.28 (−184.66–48.09)	0.249
<40	8290 ± 13,010			
40–65	3340 ± 9503.5	0.0098 *		
>65	1675 ± 6828			
Gender				
Male	2300 ± 9811	0.881 *		
Female	2300 ± 8696			
BMI, Kg/m^2^ (Spearman rho)	0.061	0.353 #		
Smoke				
Yes	1780 ± 6513	0.363 *		
No	2300 ± 9366.5			
Etiology of liver disease				
HBV	3850 ± 9444		Ref.	
HCV	1390 ± 5470		−2183.5 (−5796.3–1429.2)	0.235
MAFLD	6220 ± 11,243	0.005 *	−179.08 (−4902.54–4544.37)	0.941
ALD	2866 ± 9912		−893.91 (−9061.32–7273.49)	0.83
Autoimmune	1620 ± 9960		1318.07 (−3312.56–5948.72)	0.576
PSVD	22,200 ± 23,600		13,038.4 (3662.33–22,414.47)	0.007
Genetic disease	5254.5 ± 10,091		−1901.5 (−18,450.96–14,647.96)	0.821
HCC				
No	2619.5 ± 8936	0.652 *		
Yes	1390 ± 10,910.5			
Cirrhosis				
No	2700 ± 9222	0.089 *		
Yes	1310 ± 6486			
Child–Pugh class				
A	2490 ± 9306			
B	707.5 ± 10,510.7	0.306 *		
C	530 ± 0			
MELD (Spearman rho)	0.08	0.224 #		
Portal hypertension				
No	2619.5 ± 9280	0.206 *		
Yes	1390 ± 6518			
Liver stiffness > 10 kPa				
No	2700 ± 9279	0.167 *		
Yes	1545 ± 9226			
Immunosuppressive therapy (single drug)	1870 ± 19,835			
Immunosuppressive therapy (two drugs)	20,500 ± 0	0.316 *		
Steroids				
No	2330 ± 9360	0.256 *		
Yes	561 ± 1239			
Two or more comorbidities				
No	3110 ± 9355	0.05 *	Ref.	
Yes	1310 ± 8376		870.87 (−2413.52–4155.28)	0.602
Vaccines				
BNT162b2	2290 ± 9777		Ref.	
mRNA-1273	10,870.5 ± 14,906	0.028 *	8533.52 (3404.41–13,662.64)	0.001
ChAdOx1-S	569 ± 6000		−3851.10 (−12,249.24–4547.03)	0.367

# Spearman’s rank correlation test; * Kruskal–Wallis test. Abbreviation: CI (Confidence Interval), IQR (InterQuartile range), BMI (Body mass index), MAFLD (metabolic-associated liver disease), ALD (alcoholic liver disease), PSVD (porto-sinusoidal vascular disorder), HCC (Hepatocellular carcinoma).

## Data Availability

Data are available at University of Naples Federico II.
